# Investigating cumulative effects of pre-performance routine interventions in beach volleyball serving

**DOI:** 10.1371/journal.pone.0228012

**Published:** 2020-01-22

**Authors:** V. Vanessa Wergin, Jürgen Beckmann, Peter Gröpel, Christopher Mesagno

**Affiliations:** 1 Department of Sport and Health Sciences, Technical University of Munich, Munich, Germany; 2 School of Movement and Nutrition Science, University of Queensland, Brisbane, Australia; 3 Division of Sport Psychology, Department of Sport Science, University of Vienna, Austria; 4 School of Health and Life Sciences, Federation University Australia, Ballarat, Australia; Indiana University-Purdue University Indianapolis, UNITED STATES

## Abstract

Pre-performance routines (PPRs) can be used in certain sports to minimize the effects of choking under pressure. This study aimed to investigate the cumulative effectiveness of PPR interventions on the accuracy of beach volleyball serves. Fifty-four beach volleyball players were randomly assigned to one of three PPR intervention groups or a control group. Participants performed 10 serves at a target on the opposite side of the beach volleyball court (pretest), were educated on a PPR intervention, and then completed 10 serves at the target under pressure that was induced through videotaping and ego-relevant instructions (posttest). The results indicated no difference in post-test serving accuracy among the intervention groups and the wait-list control group and no difference in effectiveness between cumulative and isolated PPR use. A possible explanation may be the inefficiency of the pressure manipulation. However, the null results related to isolated and cumulative PPR use under general (i.e., no pressure) conditions are still an important research finding. Future research should investigate the effectiveness of cumulative and other PPRs in other sports in general and under pressure.

## Introduction

In professional and amateur sports, the pressure of competition may lead to preeminent athletes succumbing to “choking under pressure” (referred hereafter simply as “choking”), a metaphorical expression for performing worse than expected [1]. Athletes, who experience choking, are assumed either to be distracted from (e.g., [2, 3]), or too focused on (e.g., [1, 4, 5]), the execution of their action (see [6] for a review of choking theories). To improve attentional focus and thus help performance under pressure, different preparation strategies have been proposed as possible interventions (see [7] for a review on choking interventions). These interventions have been largely tested in isolation, but, recently, researchers have begun to combine the interventions and test possible cumulative effects [8, 9]. The current study investigates the inclusion of an embodiment technique, the left-hand dynamic handgrip, into broader pre-performance routines. Several studies have demonstrated that the left-hand dynamic handgrip can ameliorate or even eliminate choking under pressure [10, 11].

A *pre-performance routine* (PPR) is any cognitive and behavioral preparation strategy systematically used by an athlete prior to skill execution [12] PPR interventions typically include various elements that help the athlete adjust arousal levels when necessary (e.g., deep breathing), exert attentional control (e.g., focusing on task-relevant cues or using a “cue word” for concentration), and use behavioral steps relevant to the sport (e.g., bouncing a ball several times before a tennis serve). Psychological advantages of using a PPR may among other things constitute of lowered anxiety [13], increased task-relevant focus [12; 14], and reduced negative introspection [15]. Applied sport psychologists suggest that a PPR may be especially used in a closed, self-paced task since there is sufficient time for preparatory strategies before skill execution (e.g., [16, 17]).

Researchers have identified that performance of various skill levels can be improved through PPR use, with positive effects evidenced both in low-pressure and pressurized situations [7, 12]. Lidor and Mayan [18] in this context reported that novice volleyball players who used a behavioral PPR were more accurate in their serves than control participants who used technical instructions. Similarly, McCann, Lavallee, and Lavallee [19] found that using a PPR improved shot accuracy in novice golfers. Other researchers [16, 20] have documented the benefits of PPRs for performance under pressure. In particular, Mesagno and Mullane-Grant [20] investigated whether combining several PPR components into an extensive PPR affected performance more than the isolated use of PPR components. Mesagno and Mullane-Grant [20] found that Australian Football players using an extensive PPR showed the greatest goal-kicking accuracy under pressure, with the different preparation components in isolation showing some accuracy improvements and the control group showing slightly diminished performance. These results imply that the combined use of different elements (or preparation strategies), which interconnect well within a personalized PPR, may have beneficial performance effects.

A simple embodiment technique that has been shown to be beneficial in ameliorating performance under pressure is *left-hand dynamic handgrip* [10, 11]. Left-hand dynamic handgrip refers to clenching the left fist, or squeezing a ball in the left hand, prior to skill execution. Neurophysiological research has shown that predominant activation in the left-brain hemisphere during motor skill execution results in worsened performance [21, 22, 23]. The dominant left-hemispheric activation is most probably due to anxiety resulting from pressure, which increases a self-focus. This is associated with conscious processing during movement, which interferes with automated skill execution [23]. Left-hand dynamic handgrip activates motor centers of the right hemisphere during the hand clenching [24, 25]. Consequently, an increase in high Alpha is observed that spreads across the whole scalp [26]. High Alpha has an inhibitory function and can be said to and produces a state of cortical relaxation after the clenching. This involves a reduction of the dominant left-hemispheric activation [26]. The state of cortical relaxation after clenching the left (compared to right) hand is likely due to the higher level of connectivity and white matter in the right hemisphere (which controls the left hand), compared to the remaining parts of the brain. As a consequence, brain inhibitory mechanisms can be evoked to a greater extent in the right hemisphere when clenching the left hand [26]. Prior evidence has shown that the state of cortical relaxation is associated with enhanced readiness for motor actions [27, 28]. Based on that, Beckmann and colleagues [10] applied left-hand dynamic handgrip to optimize performance in samples of experienced athletes in soccer, taekwondo, and badminton. Results indicated that athletes squeezing a soft ball for 30 seconds with their left hand showed a better performance in a simulated competition compared to athletes squeezing the ball for the same amount of time with their right hand. These results were replicated by Gröpel and Beckmann [11] in artistic gymnastics in a real competitive environment.

It has been suggested that a combination of different interventions can have great benefits for sport performances [7] but empirical studies testing this proposition are rare. Valuable exceptions are the work by Mesagno and Mullane-Grant [20], who observed that combining different PPR elements is more effective than using the respective elements in isolation, and more recently Mesagno and colleagues [9], who combined PPRs with post-performance routines. Post-performance routines, in the Mesagno et al. study, are those mental skills that athletes use after skill execution to facilitate a return to stability and calmness of the mind. Mesagno and colleagues found that although using a post-performance routine had a positive (albeit statistically “sustained”) effect, it did not add substantially to performance effectiveness when combined with a PPR. Mesagno and colleagues [8] then combined a PPR with left-hand dynamic handgrip and tested their cumulative effect in a bowling accuracy task. Participants performed either in one of three intervention groups, consisting of left-hand dynamic handgrip only, PPR only, and combined left-hand dynamic handgrip and PPR, or in a control group. The results indicated that the single intervention groups and the combined intervention group showed the same improved performance, which was significantly better than the control group, indicating that there may not be an extra cumulative effect on performance. Hence, the recent evidence on cumulative interventions effect is scarce and mixed especially considering Mesagno and Mullane-Grant’s [20] finding that beneficial performance effects occur when combining different PPR elements, and Mesagno and colleagues’ [8, 9] results, which indicated no evidence for the cumulative performance effect.

Thus, to shed more light, this study replicates Mesagno and colleagues’[8] research by combining the left-hand dynamic handgrip with a broader PPR intervention and examines their cumulative effects on performance in beach volleyball serving. Similar to other team sports, beach volleyball performance is known to be influenced by pressure [29, 30], which makes the sport relevant for testing the above interventions as they have both been proposed to help against the performance-harming effect of pressure. Based on Mesagno et al.’s [8] results, it was hypothesized that all intervention groups would improve serving performance compared to the control group. It was further hypothesized that the combined PPR and left-hand dynamic handgrip group would not differ from the other intervention groups in their serving performance accuracy.

## Materials and methods

### Participants

An a priori G*Power calculation [31] revealed that a sample size of 48 or more participants would provide sufficient power (.80) with significant effects at an alpha level of .05. Fifty-nine experienced beach volleyball players participated in the study. Players were recruited from various volleyball clubs as well as from the Center of University Sports at the first author’s institution. Five players committed greater than six serving errors (out of the 10 serves) per session, which was more than 3 *SD*s from the sample mean. These participants were removed from the study, as their serving skills did not meet our “experienced” volleyball player sample, because being able to serve into the field is an absolute prerequisite when it comes to serving at a target. The final sample consisted of 54 participants (36 male, 18 female) aged 18 to 58 years (*M* = 27.72, *SD* = 8.28). Participants’ experience in playing volleyball varied between 3 and 35 years (*M* = 10.72, *SD* = 6.62). Twenty-nine participants currently played in a competitive volleyball league between second and 10^th^ national division in Germany, while 25 participants did not play in a league but participated frequently in collegiate tournaments. Participants were mostly inexperienced in sport psychology, whereby four participants took part in sport psychology training prior to the study. No participants (even those educated in sport psychology) had any experience with using pre-performance routines.

Prior to study participation, participants were informed about the study objectives, voluntary participation, and the confidential treatment of data. They were assured of the right to withdraw from participation at any time without consequences. All participants gave written informed consent in accordance with the Declaration of Helsinki. The study did not involve any invasive or potentially dangerous methods and therefore, in accordance with the German Research Foundation (DFG) and the guidelines of the first author’s institution, did not require formal ethical approval.

### Equipment and task

Official beach volleyball game balls (MIKASA Beach Champ VLS200) of the Fédération Internationale de Volleyball (FIVB) and standard, official 8 m (26.25 ft) x 16 m (52.50 ft) beach volleyball courts were used for the study. Following the FIVB standards, women played on a net height of 2.24 m (7.35 ft), while men played on a net height of 2.43 m (7.97 ft). A plastic pylon of 18 cm (7.09 in) diameter and 5 cm (1.97 in) height was placed on the opposing side of the court. Hereby, the center of the pylon was placed at a distance of 50 cm (19.67 in) from the sideline and the ground line. Participants chose in which corner (right or left) to serve prior to their first serve, where the objective was to serve as close to the plastic pylon as possible. After each serve, the distance between the impact point of the ball to the middle of the pylon was measured in centimeters. If the ball hit the net, it was counted as an error and the participant was allowed to repeat the serve. If the ball was hit out, it was nevertheless similarly measured since the distance from the pylon (and not the in-field hit) was the primary outcome measure. The serving errors (both nets and outs) were recorded to control for possible outliers.

### Measures

Athletes completed a demographic questionnaire that included questions related to their gender, age, years and level of experience in beach and indoor volleyball, and existing sport psychological training. To assess state anxiety, the somatic and cognitive subscales of the Mental Readiness Form-3 (MRF-3; [32]) was used. The MRF-3 subscales consisted of three 100 mm continuums: cognitive anxiety ranging from *calm* to *worried*, somatic anxiety ranging from *relaxed* to *tense*, and self-confidence ranging from *confident* to *not confident*. The MRF-3 allows participants to indicate their current condition quickly by placing a mark on each of the three lines. The distance between the left end of the line and the participants mark constituted the participants’ scores. The scores could range from 0 on the left end to 100 on the right end of the line, whereby higher scores indicated higher anxiety. Similar to Mesagno and colleagues’ [8] study, only the somatic and cognitive subscales of the MRF-3 were used, as these contain the central aspects of anxiety.

### Experimental groups

Participants were randomly assigned to one of the three intervention groups (PPR, left-hand dynamic handgrip, combined PPR and left-hand dynamic handgrip) or to a wait-list control group. Participants in the PPR group developed a PPR together with the first author, who is an experienced volleyball and beach volleyball player and a certified sport psychologist. The PPR was individualized (i.e., different PPR elements used depending on what the first author thought was important) for each participant and therefore varied in type and number of applied PPR elements. Although there was some PPR variability among participants, a general template of elements was offered to all participants, which helped to maintain robust experimental research design standards. Possible PPR elements included the adjustment of arousal levels through deep breathing, behavioral steps (e.g., rotating the ball in both hands), and attentional control (e.g., focusing on the ball hitting the target pylon), which were PPR elements efficiently used in other PPR studies (e.g., [20]). The first author supported all athletes in developing their routines by watching their current, mostly automatic, behaviors prior to a serve and suggesting additional one or more elements in consultation with the athletes. For example, if athletes were observed to take a long look at the ball before serving, they were asked to first focus their attention on the ball by looking at it, then on the target, also by looking at the target, and to take a deep breath before serving the ball. Participants were given approximately 10 minutes to practice their routine, prior to the start of data collection.

Participants assigned to the left-hand dynamic handgrip group squeezed a soft “anti-stress” ball in their left hand twice a second for 10s before each serve. The duration of 10s was chosen to be consistent (and comparable) with the duration of Mesagno et al.’s [8] study. The combined PPR and left-hand dynamic handgrip group first completed the 10s of dynamic handgrip followed by the execution of their PPR. The PPR was always performed after the dynamic handgrip since it contained elements that led into the service execution. The wait-list control group did not receive any PPR education.

### Procedure

The study was performed on a regular beach volleyball court. Participants were recruited from various volleyball clubs around Munich, Germany, and from the University Sports Center. Before commencement of the experiment, volunteer participants completed the demographic questionnaire and warmed up until they indicated their readiness to start serving.

Participation took place individually and included a performance accuracy task before (called pretest), and after (called posttest), the implementation of the PPR, left-hand dynamic handgrip, or combined interventions. The pretest and posttest took place on the same day with the intervention development and implementation session separating them. In the pretest, participants took two practice serves at the target pylon followed by another 10 recorded serves. Participants were offered 20–30 seconds between serves for time consistency among participants. This time allocation was enough time for the researcher to measure the volleyball accuracy, record the score, return the volleyball to the participant, and allow the participant to set up again for the next attempt. After two (of the 10) recorded serves, participants completed the MRF-3 to report their state anxiety and then performed the remaining eight serves. A research assistant measured the distance between the impact point of the ball and the target on the court.

After the pretest serves were completed, the intervention groups received a group-specific intervention development and implementation training session. Similar to participants in the Mesagno et al. [8] study, participants practiced their “newly” developed intervention in two practice “shadow” serves (i.e., completing the steps without the use of the ball) and until the first author and the participant were satisfied that the participant understood the intervention. After that, the posttest was conducted, which only differed from the pretest regarding the pressure induction and regarding the intervention implementation for participants of the three interventions groups before each serve. Like in the Mesagno and colleagues [8] study, a video camera was set up next to the volleyball court, in front, and in full view, of the participant to induce pressure. Each participant was told that the current beach volleyball study would be broadcasted on regional media because of the collaborative research project being undertaken between the two internationally recognized universities. In addition to the elements of the pressure induction used by Mesagno and colleagues [8], participants were asked to introduce themselves to the camera and state how long they had been playing beach volleyball before starting with the posttest serves in order to further increase their anxiety level and perceived pressure. Next, participants performed the posttest serves until the serves were completed with the video camera turned on and facing them the entire time. Control group participants performed the serves similar to the pre-test, while intervention group participants performed their group-specific routine after the ball was returned and before the next attempt. After being debriefed about the design of the study, including the pressure manipulation through cameras and the pretense of a TV broadcast, participants were thanked and dismissed.

## Results

### Homogeneity of groups

A one-way Analysis of Variance (ANOVA) on pretest performance indicated no significant Group differences, *F*(3, 50) = .24, *p* = .87, η_p_^2^ = .01. Furthermore, a one-way ANOVA on experience level in years indicated no significant Group differences, *F*(3, 49) = 1.93, *p* = .14, η_p_^2^ = .11. Thus, equal group-based serving accuracy ability and experience was assumed.

### State anxiety

Means and standard deviations of cognitive, and somatic, anxiety for the different groups are presented in Table 1. Two separate 4 x 2 (Group x Phase) repeated measures ANOVAs were conducted for the two anxiety subscales for the pretest and posttest phases. Both analyses indicated no phase main effects (*F*s < .36; *p*s > .55), no group main effects (*F*s < 1.14; *p*s > .34), and no interactions (*F*s < .47; *p*s > .70), indicating that participants cognitive, and somatic, anxiety did not change from pretest to posttest.

**Table 1:** Means and standard deviations in parentheses for cognitive anxiety, somatic anxiety, and accuracy (Mean Absolute Distance—MAD) pre- and post-intervention for the different groups.

Group	Cognitive Anxiety		Somatic Anxiety		Accuracy (MAD)	
	Pre	Post	Pre	Post	Pre	Post
PPR	22.21 (16.11)	25.36 (15.27)	30.00 (19.30)	32.71 (19.54)	168.41 (40.64)	156.41 (36.30)
Dynamic handgrip	15.38 (13.07)	18.62 (12.47)	24.62 (19.74)	24.00 (15.76)	165.89 (48.49)	145.28 (31.38)
Combined	24.08 (18.90)	25.42 (14.31)	35.08 (14.69)	33.42 (20.38)	155.48 (34.04)	147.29 (47.12)
Control	24.40 (21.67)	25.73 (18.85)	29.87 (21.19)	29.13 (19.05)	158.58 (51.61)	165.26 (47.20)

### Task performance

Means and standard deviations of volleyball serve accuracy for the different groups are presented in Table 1. A 4 x 2 (Group x Phase) repeated measures ANOVA was conducted to examine volleyball serve accuracy during pretest and posttest. The analysis indicated neither a phase main effect, *F*(1, 50) = 2.14, *p* = .15, η_p_^2^ = .04, nor a group main effect, *F*(3, 50) = .27, *p* = .85, η_p_^2^ = .02. Furthermore, there was no interaction, *F*(3, 50) = 1.01, *p* = .40, η_p_^2^ = .06.

## Discussion

The aim of the current study was to test the cumulative (i.e., additive) effects of an embodiment technique (left-hand dynamic handgrip) and a broader PPR intervention on performance. This study essentially replicated the PPR interventions tested by Mesagno et al. [8] in tenpin bowling but instead used these routines for improving accuracy of a beach volleyball serve under pressure (albeit with some minor pressure manipulation modifications). In accordance with the results of Mesagno and colleagues, it was expected that the intervention groups would perform more accurately than the wait-list control group from pretest to posttest (under pressure). We, however, found no evidence for the effectiveness of PPRs in this volleyball-serving task.

### Pressure manipulation

Similar to the results of Mesagno and colleagues [8], the pressure induction in the current study was not effective, as it did not significantly increase anxiety in players. The pressure manipulation in the current study was similar, but not identical to, the pressure manipulation in the Mesagno et al. study. We hoped that a slight variation of asking participants to give a brief “impromptu” speech to the video camera prior to the posttest serves would make the collaborative research project instructions more believable and anxiety provoking in the current study, which was not the case. Thus, an improving effect of PPRs on performance under pressure that has been reported in many other studies (e.g., [16, 20, 33]) could not be investigated in the current study.

Reasons for the ineffectiveness of the pressure induction may be that many participants of the current study were playing in volleyball clubs or leagues, where video recording is common during practice and competitions. Participants might have become accustomed to being filmed while performing. In future studies, a more effective method (or a combination of methods) of pressure induction should be applied, such as simulation of a competitive situation or the presence of audience [7]. In light of the ineffective pressure manipulation, the independent and cumulative PPR effects are discussed under general (i.e., no pressure) conditions since this adds to the current literature.

### Independent PPR effects

No differences in performance accuracy emerged among intervention groups and control group from pretest to posttest. Thus, independent (i.e., extensive or single element) PPRs were not effective in improving participants’ serving accuracy in the current study after a 10 min routine development session. Similar null results of independent PPRs have been reported infrequently [9, 13], while most published studies suggest the effectiveness of independent PPRs in improving performance accuracy (see [12] or [7] for an overview). Our results are hence at odds with the majority of reported findings that support individual PPR intervention use (e.g., [34, 35, 36]).

Many explanations for our null effect for the independent PPR effects may be offered, but we provide two. First, beach volleyball in its nature is different from “pure” accuracy sports such as tenpin bowling or golf. While accuracy represents the essence of these sports, serves in beach volleyball constitute only one aspect of the sport and are therefore less often practiced than, for example, a typical shot in ten-pin bowling. In beach volleyball, other components, including blocks, attacks, reception, and defense, play an important role for a successful game as well [37]. In fact, besides serving effectiveness, attack efficiency is a main factor for winning a beach volleyball game [38]. Moreover, the accuracy of the serve is only one component influencing the overall serving success. Speed and flight curve [37] constitutes another, maybe even more important, component of the serve. If a player is capable of serving a fast or floating ball, accuracy of the serve does not play such a significant role because the opponent will struggle with the serve independent from its placement on the court. Thus, the effectiveness of PPR interventions for serving accuracy in beach volleyball may be limited, because it is a combination of several serve components rather than perfect accuracy alone. Second, the simple 10-minute routine development session may not have been long enough to see positive performance effects. Although the current study routine development was designed specifically for the pressure manipulation in mind (i.e., to encourage appropriate task-relevant attention prior to execution under pressure), other researchers [9, 13] who provided longer routine “acquisition” times (e.g., 7 days to 4 weeks) to develop and embed PPR learning also found null results for independent routines. Perhaps future research could examine a dose effect of routine development length to determine the best amount of time for athletes to spend developing and practicing a routine for improved performance. Nevertheless, this is the first known study to show no performance effects after a short (i.e., less than 30 min) PPR development period.

### “Cumulative” effects of the PPRs

We tested whether the combination of left-hand dynamic handgrip, which had been proposed to ameliorate self-focus [10, 11], and a broader PPR intervention, proposed to prevent distraction [20], have cumulative effects on performance. Results showed no differences between the combined PPR and the isolated PPR use. Thus, cumulative effects of the combined PPR intervention on performance accuracy cannot be supported. A few existing studies do report an advantageous effect of an extensive PPR compared to isolated, single element PPR use (e.g., [20]), while other researchers [8, 9] did not find any cumulative effects of combined PPR interventions. Our results are in line with the latter evidence showing that aggregating different interventions does not automatically translate into a cumulative benefit. This implies that providing an athlete with a single, easy-to-understand, and individualized PPR is a good option in applied settings with limited time for intervention development because it is likely to give the athlete a similar advantage as an extensive PPR

These disparate results for cumulative effects of combined routines may be a product of methodology [8] and the type of combined routine used. For example, Mesagno and Mullane-Grant [20], similar to the current study, used a short intervention development session, but included the combination of elements that focused attention on task relevant cues prior to performance execution only, which led to improved performance for the extensive PPR. The Mesagno et al. [9] study, however, asked participants to follow a combined routine that included elements prior to, and then after, shot execution and allowed a 4-week routine education period. Furthermore, Mesagno et al. [8] used a short intervention development session and routine elements that were prior to execution only, similar to the current study, but also argued that the cognitive overload of the left-hand dynamic handgrip and PPR may have been uncomfortable for participants, which null effects. Thus, perhaps combining the different methodologies and combined routines may provide an avenue for future research. That is, perhaps investigate the cumulative effects of combined PPR interventions that emphasize routine elements prior to shot execution only with longer routine development periods.

### Limitations and future research

Although we attempted to provide a robust design, there are limitations that may have affected our results. First, the PPR and left-hand dynamic handgrip interventions were new to participants, and thus some participants might have been overwhelmed by the different elements of the PPR, especially in the combined intervention group. Future researchers should provide more time to practice a combined PPR intervention in order for participants to become more familiar with it (especially considering other researchers informal findings [9] indicated that participants were distracted by the routine in the early routine development stages), which might increase its effectiveness [16]. Second, we did not control for other important components of beach volleyball serving (speed, flight curve), which should be investigated in future studies. Third, we only used videotaping (with a brief speech prior to serves) to induce pressure. Although videotaping had been found to manipulate pressure previously [39], recent studies have found this pressure-inducing method less effective, presumably because athletes get increasingly used to it [40, 41]. Researchers investigating performance under pressure should therefore combine different methods (e.g., simulated competition, the presence of audience, ego relevance) to effectively increase pressure [7]. Finally, our sample consisted of volleyball players of many different competitive levels (from the 2^nd^ German league to collegiate tournaments). Even though there were no differences in serving accuracy among the various competitive levels, which was presumably due to the small number of participants within the levels, future research could beneficially use more homogeneous samples to more reliably evaluate the effectiveness of PPR interventions.

## Conclusions

This study provided first insights into the effectiveness of a PPR and the left-hand dynamic handgrip on serving accuracy in beach volleyball. Results indicated no improvement in serving accuracy related to the isolated use of PPR and left-hand dynamic handgrip interventions. Combining PPR and dynamic handgrip interventions did not lead to benefits in performance accuracy compared to their isolated use. To shed more light onto the consistency of these interventions’ effectiveness in various sports, future research should address different types of individual and team sports and investigate the interventions without and under pressure using ecologically valid testing and pressure induction.
